# Perspective: Structure and ultrafast dynamics of biomolecular hydration shells

**DOI:** 10.1063/1.4981019

**Published:** 2017-04-20

**Authors:** Damien Laage, Thomas Elsaesser, James T. Hynes

**Affiliations:** 1 Ecole Normale Supérieure, PSL Research University, UPMC Univ Paris 06, CNRS, Départment de Chimie, PASTEUR, 24 rue Lhomond, 75005 Paris, France; 2 Max-Born-Institut für Nichtlineare Optik und Kurzzeitspektroskopie, 12489 Berlin, Germany; 3 Department of Chemistry and Biochemistry, University of Colorado, Boulder, Colorado 80309, USA

## Abstract

The structure and function of biomolecules can be strongly influenced by their hydration shells. A key challenge is thus to determine the extent to which these shells differ from bulk water, since the structural fluctuations and molecular excitations of hydrating water molecules within these shells can cover a broad range in both space and time. Recent progress in theory, molecular dynamics simulations, and ultrafast vibrational spectroscopy has led to new and detailed insight into the fluctuations of water structure, elementary water motions, and electric fields at hydrated biointerfaces. Here, we discuss some central aspects of these advances, focusing on elementary molecular mechanisms and processes of hydration on a femto- to picosecond time scale, with some special attention given to several issues subject to debate.

## I. INTRODUCTION

The water shell around biomolecules can exert a strong influence on their structural and functional properties.^1–3^ Accordingly, much effort has been expended on obtaining structural information about these molecules and their hydration shells. As is well known, biomolecular equilibrium structures have been unraveled with highly sophisticated methods of structure research such as x-ray diffraction from crystallized samples^4–7^ as well as x-ray and neutron scattering from solid or liquid samples without a long-range atomic order. While the focus of x-ray diffraction has primarily centered on biomolecular atom positions, some limited information on the locations of water molecules in the crystal could be obtained. In both x-ray diffraction and scattering, water locations have been traced via their oxygen atoms' positions, but the hydrogens' positions have remained elusive because of their small scattering amplitude due to their low electron density. Fortunately, insight into the hydrogen atoms' spatial pattern can be obtained from neutron scattering.

Such methods have generated many impressive results, but there are difficulties to be overcome. Extrapolation from the crystalline phase and/or model systems of reduced complexity to biomolecules in an aqueous environment at ambient temperature is far from being straightforward, to say the least. Other major issues are the strong excess of water, i.e., the water shell's much larger extension in the liquid environment, its structural heterogeneity on passing from the first water layers around the biomolecule on into the bulk, and the fact that both the biomolecule and its water shell undergo structural dynamics on a multitude of time scales; indeed, the water shell dynamics are a key and highly relevant dimension not addressed by classic structural studies. This challenging complexity calls for a concerted combination of experimental and theoretical methods which address the dynamics of structures and interactions and allow their elucidation in time and space.

Three particular aspects of the hydration of biomolecules have attracted strong interest: (i) To what extent and over which range is the water structure affected by a biomolecule's presence? Compared to the bulk liquid in which each water molecule has close to four neighboring waters connected by hydrogen bonds, at the heterogeneous interface with the biomolecule, the spatial arrangement of water molecules and their interactions are obviously modified. The spatial range of this modification when moving away from the interface has, however, remained a highly controversial issue and a topic of current research (see, e.g., Refs. 8 and 9). (ii) Related to this is the question: To what degree and in what fashion are water dynamics in a hydration shell different from bulk water? In the bulk liquid, the thermally excited structure fluctuations of water imply librational motions on a subpicosecond time scale and a stochastic breaking and reformation of intermolecular hydrogen bonds on the time range of a few picoseconds. Given the steric constraints set by the biomolecular surface, the water dynamics in the first few hydration layers may differ substantially from those in the bulk. If the shell waters were much slower than bulk water, rearrangements within the shell could become rate-limiting for some key functional processes in the biomolecule. (iii) Numerous biomolecules contain ionic and/or highly polar groups, displaying Coulomb interactions among each other and with the water dipoles in the environment. Neither the strength nor the range of these interactions is well understood.

**FIG. 1.**
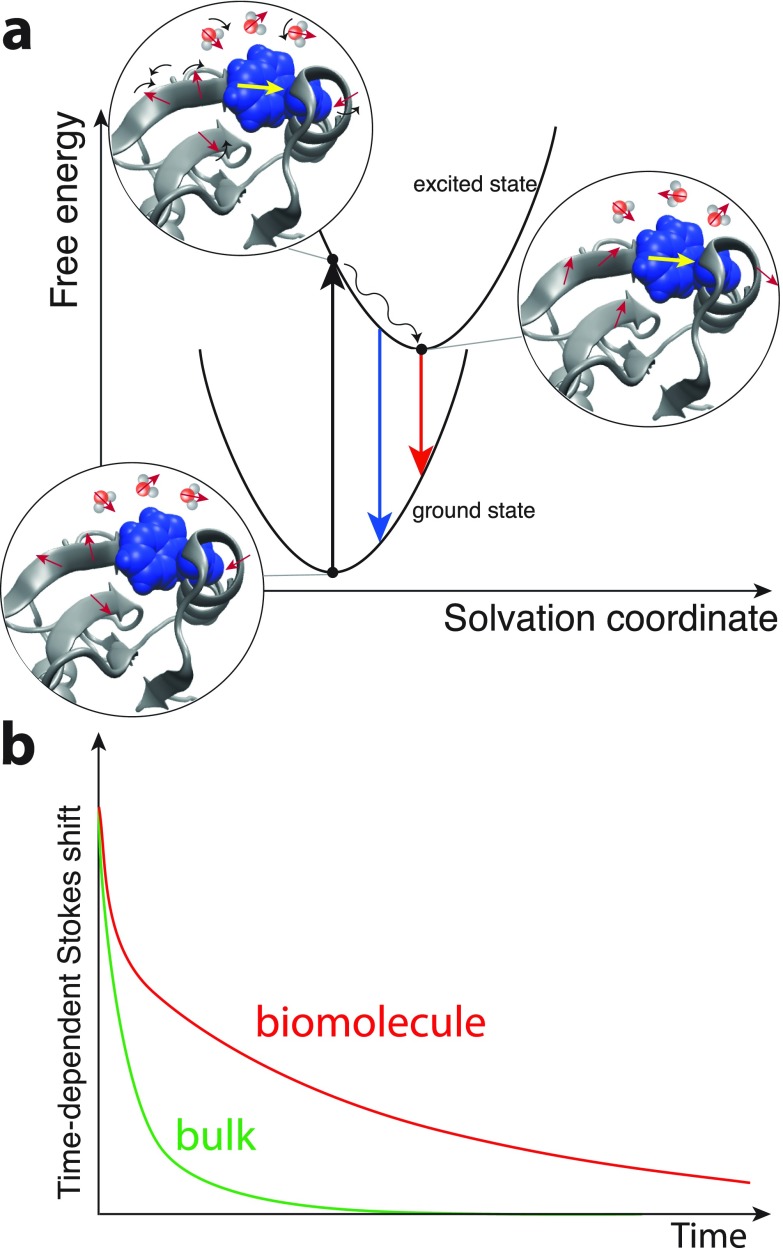
Schematic description of the time-dependent Stokes shift (TDSS) measurement of solvation dynamics.^10^ (a) Electronic ground and excited state free energy surfaces of the chromophore attached to the biomolecule along an energy gap, or generalized solvation coordinate that characterizes the probe's electrostatic interaction with its environment, consisting (primarily) of the solvent and the polar and charged sites on the biomolecule. (b) Typical time-dependent Stokes shift decay in time for the chromophore in bulk water and attached to the biomolecule.

Ahmed Zewail and his co-workers have pioneered Time-Dependent Stokes Shift (TDSS) studies of fluorescence or stimulated emission from an organic chromophore attached to or incorporated into a biomolecule's structure (Fig. 1).^10–12^ An ultrashort optical pulse promotes the chromophore to an electronically excited state and the emission spectrum's temporal evolution is mapped via a time-resolved detection scheme, e.g., fluorescence up-conversion, or by inducing stimulated emission with a probe pulse. The emission undergoes a red-shift due to solvation, i.e., the interacting environment of the probe adjusts in response to the chromophore's dipole change, thus lowering the excited state free energy. In the limit of a linear response, the time-resolved normalized Stokes shift is proportional to an electronic state energy gap time correlation function, whose dependence on the environmental dynamics can reflect the important components of the ensemble-averaged dynamics of the hydration shell and other features of the probe's environment. Zewail and co-workers have applied this method to a variety of proteins, DNA, and other biomolecular systems;^10,13–16^ they have found kinetics on various time scales, sometimes extending to hundreds of picoseconds, much slower than solvation processes induced with the same chromophores in bulk liquid water. They interpreted the latter results in terms of a substantial slowing down of water dynamics around biomolecules, in their view a characteristic of “biological water.”^11,12,16^ This echoed prior studies which had similarly concluded that shell dynamics are much retarded with respect to the bulk.^17,18^

**FIG. 2.**
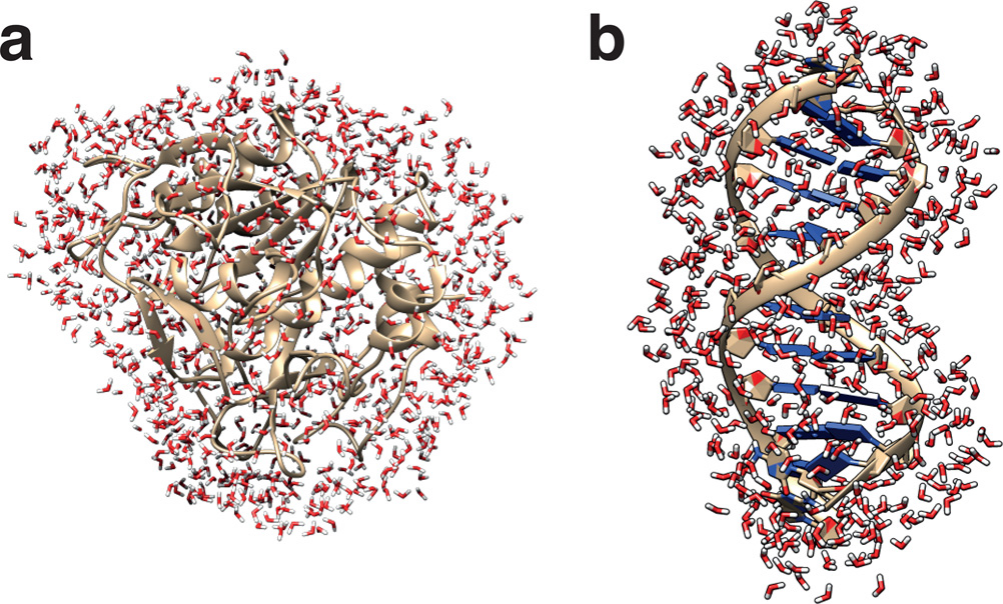
First hydration shells of a protein (a) and of a DNA dodecamer (b).

This and other work has been succeeded by extensive research in biomolecular dynamics, both experimental, e.g., via methods of nuclear magnetic resonance (NMR) and/or ultrafast vibrational spectroscopy, and by theoretical and simulation studies. In this article, we discuss results from recent research to address—and to put into perspective with earlier work—several key features of structural aspects and time scales of biomolecular hydration. A critical assessment of experimental methods is combined with a discussion of the basic molecular mechanisms and interactions governing structure and dynamics of such highly complex systems. We first address hydration dynamics on femto-to picosecond time scales by introducing theoretical concepts and experimental probes in Sections II A and II B. This is followed by a description of equilibrium structure and fluctuations (Section II C) and a discussion of elementary processes at the molecular level (Section II D). Concluding remarks are presented in Section III. Our presentation here is of course limited in extent and scope; for a much more complete and extensively referenced discussion of these and allied issues, we refer the interested reader to Ref. 19 by the present authors.

## II. HYDRATION DYNAMICS ON A FEMTO- TO PICOSECOND TIME SCALE

### A. Hydration layer and simulations

Clearly separating the hydration shell from the bulk is not trivial. The hydration shell is usually defined (with the intent) to include all those water molecules whose local environment, interactions, and dynamics are affected by the biomolecule's presence (Fig. 2). However, the practical implementation of this intuitive definition faces several difficulties. For example, the hydration shell's contours may depend on the structural or dynamical observable being considered, the intensity of the biomolecule's effect may fade away progressively rather than abruptly with the distance to the biomolecule's interface, and finally the hydration shell is not a homogeneous ensemble: the magnitude of the effect induced by the biomolecule can be more or less pronounced in different parts of the shell.

Molecular dynamics (MD) simulations represent a key method for describing hydration shell structure and dynamics (although subject to the quality of the force fields employed). The shell is typically defined with the help of geometric criteria, e.g., the distance *d* between the water molecule's oxygen atom and the closest biomolecule atom. The first layer of water molecules directly in contact with the biomolecule can thus be selected by using a typical value for *d* of approximately 3.5 Å. However, variations in the first shell thickness result from hydrophobic sites which tend to repel water molecules, and from hydrophilic—especially charged—sites which tend to attract them. In any case, the hydration shell thickness around a given site can then be determined as the distance to the first minimum in the radial distribution function (rdf).

**FIG. 3.**
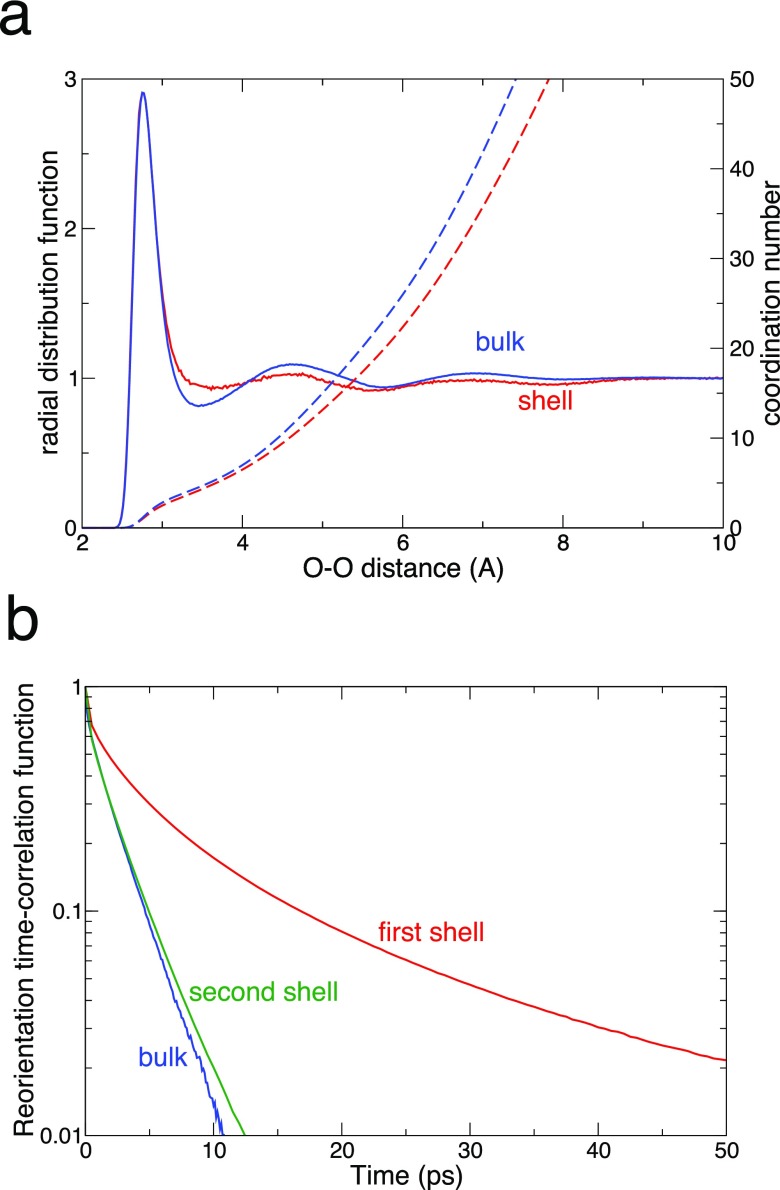
Comparison of structural and dynamical properties of a biomolecular hydration shell and bulk water. (a) Water oxygen-oxygen radial distribution functions (solid lines) and coordination numbers (dashes) for a water molecule lying, respectively, in the first hydration shell of a DNA dodecamer^21^ (red) and in the bulk (blue). (b) Water second-order reorientation time-correlation functions for a water molecule initially lying in the bulk (blue) and in a DNA dodecamer's first (red) and second (green) hydration shells.^21^

The time-averaged distribution of water molecules from MD simulations allows for generating rdfs which can be compared directly to radial distributions derived from structure-sensitive experiments. Beyond such static measures, a broad range of dynamical properties can be calculated for the hydration shell's water molecules, including the rotational, translational, and hydrogen-bond dynamics, the residence time—i.e., the time spent by a water molecule in the hydration shell before leaving into the bulk—and various experimentally accessible dynamical quantities. Indeed, numerical simulations have been extensively applied to, e.g., protein and DNA hydration dynamics. Such simulations have shown that the effect of the biomolecule on these dynamical properties is essentially limited to the first few hydration layers^20,21^ (Fig. 3), thus both justifying the above-described geometric definition selecting the first hydration shell and countering claims of significantly longer-ranged influence. But it is important to remark that determining the first shell's dynamical properties faces an additional difficulty due to the frequent exchanges between the shell and the bulk; considering only those water molecules that continuously reside within the first shell artificially focuses on a small fraction of very slow molecules and is likely to lead to artifacts.^22^

### B. Experimental probes

The impact of a biomolecule on the surrounding water dynamics is characterized by both the number of water molecules whose dynamics differ from those in the bulk and the magnitude of this effect on each of them. However, there is presently no single experimental technique able to unambiguously determine both aspects.

Beyond the methods mentioned in the Introduction that provide time-averaged structures, a broad range of further experimental techniques give insight into hydration shell *dynamics*. These techniques probe different, complementary aspects of the dynamics; further, these dynamics are not necessarily affected in the same way or to the same degree by the biomolecule. Some techniques address different types of motions, e.g., translational, rotational, and vibrational dynamics (neutron scattering,^23,24^ NMR,^25,26^ and femtosecond infrared spectroscopy^27^); some are sensitive to the averaged motions of individual water molecules—e.g., NMR and femtosecond infrared—while others probe more collective rearrangements, i.e., macroscopic observables are measured to which many water molecules contribute—e.g., dielectric relaxation, THz, and optical Kerr-effect spectroscopies.^28–31^ It is relevant to stress that gaining a molecular-level insight into these collective distortions of the hydrogen-bond network from frequency-domain spectra requires spectral deconvolutions which rely on (sometimes rather strong) assumptions about and (sometimes rather simplified) models of the ensemble's response to the external stimulus.

All these experimentally measured quantities are averaged over the molecular ensemble, resulting in a loss of spatial selectivity, i.e., water molecules close to and far away from the biomolecule are difficult to distinguish. To circumvent this problem, some experiments have been performed with a high biomolecular concentration so that essentially each water molecule belongs to a hydration shell. The very significant price to pay is that this hydration pattern is quite different from diluted samples, and the sharing of waters between biomolecules can affect the dynamics substantially.

Other experiments probe water dynamics less directly: they are based on the introduction of spectroscopic probes which are sensitive to equilibrium fluctuations and/or solvation and relaxation processes in the hydration shell. The TDSS method introduced by Zewail and co-workers^16^ is prototypical for this approach; other more recent examples are femtosecond pump-probe and two-dimensional (2D) spectroscopy of vibrational probes in the electronic ground state.^19^ Vibrational spectroscopy probes interactions and dynamics via changes in the spectral position and/or lineshape or, in the case of 2D spectroscopy, via the 2D signals' spectral shape and time evolution.^32,33^ Caution is required here: introduction of additional chromophores for TDSS measurements and/or additional vibrational probe molecules changes the hydration pattern around the biomolecule (and can even alter the local biomolecular structure); this invasive character of the probes must be accounted for when interpreting the results. Alternative non-invasive probes are vibrations of the biomolecule which are located at its interface with the water shell and, thus, are particularly sensitive to water dynamics in the structurally unchanged hydration shell.^34^ In general, spectroscopic probes are subject to interactions with all the system's constituents, including not only the water shell but also the biomolecule and, if present, its counterions. This complicating but sometimes forgotten fact presents a challenge for interpreting the time evolution mapped by the probe, in particular on longer pico- to nanosecond time scales where the biomolecule's motions will come into play.

We note that the different experimental methods described above cover different aspects of hydration dynamics in a time range extending from some 50 fs into the millisecond domain. Depending on the measured quantity, they probe the first few water layers around the biomolecule up to a large volume in space without providing direct structural information. While none of these methods allows for a clear determination of the number of water molecules affected by the presence of the biomolecule, they have considerable potential for unraveling the average molecular interactions underlying the dynamical behavior of biomolecules and water shells.

### C. Equilibrium structure and fluctuations

In this subsection, we first consider the hydration shell's time-averaged equilibrium structure and then discuss the time scales of structural fluctuations in the thermal equilibrium ground state of a hydrated biomolecule. Clearly, the structure and chemical properties of a protein and/or DNA surface are highly heterogeneous and define a complex pattern of boundary conditions for water molecules in the biomolecule's first layer, whose impact can extend beyond that layer. There are steric constraints and different interactions—hydrogen bonds, hydrophobic forces, and long-range Coulomb forces from charged or highly polar groups—all influencing the spatial arrangement of the water molecules. As a result, the structure of the first water layer displays a distribution of local geometries which are distinctly different from the molecular arrangements in bulk water. Three prominent examples are the “spine of hydration” in the minor groove of DNA,^5^ which is a chain-like arrangement of water molecules, the hydration shells around phosphate groups,^35^ which consist of up to 6 water molecules in the first layer, hydrogen-bonded to the free ${\text{PO}}_{2}^{-}$ oxygens, and the clathrate water structures around hydrophobic groups.^36^

For a sufficient dilution of biomolecules in the aqueous phase, the neighboring water will assume the structure of the bulk liquid with increasing distance from the biomolecule's surface. The range, or number of water layers over which this happens, has remained somewhat controversial. Radial distribution functions of water oxygens derived from MD simulations suggest a narrow range of a few (≃3) layers over which the bulk water structure is established (cf. Fig. 3(a)). This conclusion is supported by theoretical and experimental studies of water nanopools confined in reverse micelles with sulfonate or phosphate headgroups.^37,38^ For nanopool radii of some 10 Å and larger, one observes a bulk-like water core in the center of the nanopool with structural and dynamical properties close to bulk water and distinctly different from the few interfacial water layers.^38^ In contrast to this limited range, terahertz studies which have addressed intermolecular water modes in hydration shells under quasi-stationary conditions have been interpreted in terms of a dynamical distortion extending over a significantly larger range, e.g., distances of more than 20 Å from a protein's surface.^29^ This conclusion has been drawn on the basis of a number of simplifying assumptions regarding the distribution of proteins in the solution and the water shell's dynamical properties,^39^ and so should be regarded with care. In this connection, we recall that steady-state spectroscopy probes the linear dielectric function but does not entail structure information in a direct way.

**FIG. 4.**
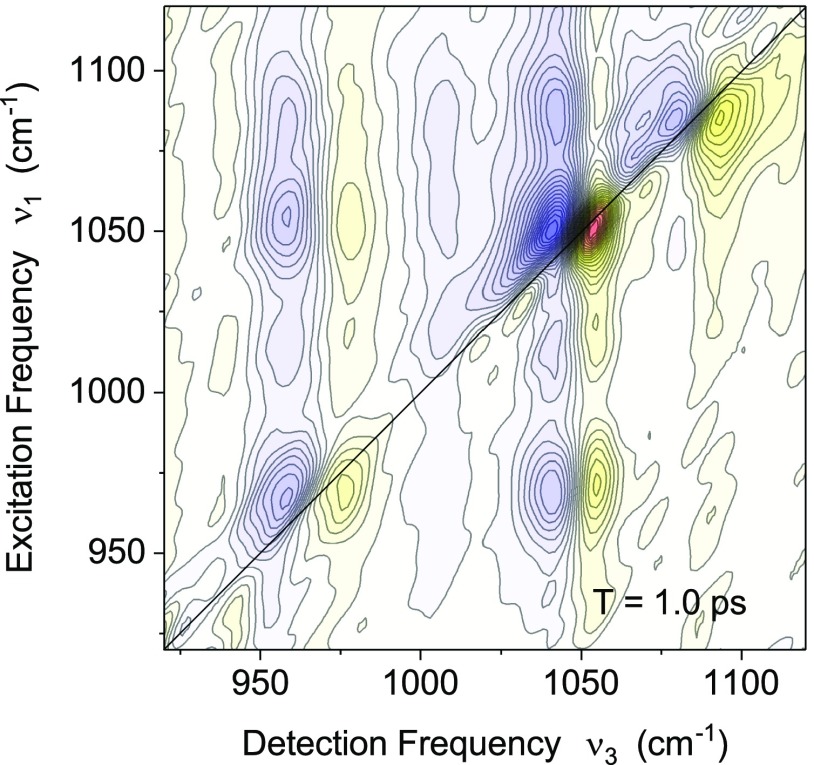
Two-dimensional infrared spectrum of backbone modes of salmon testes DNA in aqueous solution. The absorptive 2D signal is plotted as a function of the excitation frequency *ν*_1_ and the detection frequency *ν*_3_. Red yellow contours are positive signals and blue contours represent negative signals. The signal changes by 5% between adjacent contour lines. The diagonal peaks ($\nu_{1}=\nu_{3}$) represent the response of the individual backbone modes with positive signals from their v = 0 to 1 and negative signals from the v = 1 to 2 transitions. The lineshapes of the positive diagonal peaks were used to extract frequency fluctuation correlation functions.^32^ The cross peaks are due to intermode couplings and population transfer between the modes.

The thermally excited motions of water and biomolecules give rise to structural fluctuations on a multitude of time scales.^19^ The fastest fluctuations occur on a femtosecond time scale and have mainly been probed by femtosecond vibrational spectroscopy, in particular by two-dimensional (2D) infrared spectroscopy and pump-probe methods, including measurements of pump-probe anisotropies.^40^ These techniques cover a time range determined by the vibrational excitations' lifetimes, a range up to some 10 ps at most. In 2D spectroscopy, the loss of correlation between the vibrational transition frequency at early and later waiting times gives rise to a reshaping of the 2D lineshapes mapped in the experiment.^41^ Their analysis allows retrieval of the ensemble's frequency fluctuation correlation function (FFCF)—characterizing the dynamics of the loss of the frequency memory—and its comparison with predictions from and interpretations given by theory and MD simulations.

A first group of studies has focused on OH stretch excitations of the water shell at different hydration levels, i.e., water content of the samples. For a large water excess, the measured 2D spectra are averaged over water molecules both next to and far away from the biomolecule's surface; at reduced hydration levels, waters predominantly close to the biomolecule are probed instead. For water shells around DNA, phospholipids and selected proteins, a gradual FFCF decay with time constants between 300 and 500 fs has been found, which is somewhat slower than the sub-100 fs initial decay observed for bulk water.^42^ Additional decay components are observed in the picosecond time domain, which are frequently superimposed on a residual quasi-constant background which reflects inhomogeneous broadening of the vibrational lineshapes.

The 2D infrared spectra of interfacial probe vibrations—such as the phosphate vibrations of phospholipids and DNA and other backbone vibrations of DNA (Fig. 4)—give complementary and more specific insight, since they predominantly probe the first few water layers and fluctuations of the biomolecule itself. For both fully hydrated phospholipid head groups in reverse micelles^43^ and fully hydrated DNA,^32,34,44^ the initial 300 fs decay of the FFCF is followed by a long-lived contribution with a decay time longer than some 10 ps. The finding of a similar FFCF for DNA with only two complete hydration layers suggests that the fluctuating forces which govern the FFCF of interfacial vibrations originate mainly from the biomolecule's first few water layers (to be discussed in more detail in Section II D 1). MD simulations of spectral diffusion of the asymmetric phosphate stretch vibration in hydrated DNA give a similar FFCF behavior.^45^ Such slowing-down of the interfacial water has been rationalized by invoking the impact of the steric restrictions imposed by the corrugated biomolecular surface on librational and other intermolecular motions.

Slower processes resulting in structure fluctuations can occur in the time range from a few picoseconds up to nanoseconds. Water molecules may exchange their hydrogen-bond partners, reorient, and leave the hydration shell. Two important reference times here are, for the dynamics in the bulk, 2 ps for the reorientation time of a water molecule, and second, for the biomolecule's own dynamics, typical nanosecond tumbling times. Reorientation processes of water molecules and—to lesser extent—smaller functional units of biomolecules have been studied in magnetic relaxation dispersion,^25^ and in polarization-resolved pump-probe experiments, providing the ensemble-averaged anisotropy decay of vibrational dipoles.^40^ The measured reorientation dynamics of water molecules in hydration shells cover a time range from a few to tens of picoseconds. This demonstrates only a moderate slowing down of the intrinsic dynamics of the hydration shell water compared to bulk water; this issue is further pursued below. In view of such results, the concept of an extremely retarded hydration shell potentially hindering the biomolecular conformational rearrangements, as described in Section I, has to be discarded.

### D. Time scales of biomolecular hydration dynamics

A number of molecular interactions and mechanisms determine the hydration structure and dynamics at a biomolecular surface and in the water shell. Intermolecular couplings through hydrogen bonds, electrostatic forces from charged and polar groups, and hydrophobic forces are of similar and limited strength, resulting in the heterogeneous and highly complex dynamic behavior of the molecular ensemble. In the following, we focus on some key aspects which have been addressed by theory and experiment and we successively examine the different time scales of biomolecular hydration dynamics (which are sometimes controversial).

#### 1. Electric field fluctuations and ultrafast dynamics

The dipolar character of water molecules gives rise to strong electric fields acting within the hydration shell and at its interface with the biomolecule. Charged and/or polar units of the biomolecule are additional sources of electric fields. At ambient temperature, the fluctuations in molecular geometry and spatial arrangement result in electric-field fluctuations on a multitude of time scales. For an adequate theoretical description and experimental characterization of this complex many-body system, one has to address the frequency spectrum, strength, and spatial range of electrostatic forces, as well as these forces' variation with distance from the biomolecular surface and influence on structure and functional processes. In theory and simulation, such issues have been treated at different levels of sophistication, ranging from simple static Poisson-Boltzmann calculations to molecular dynamics simulations including polarizable molecular units and water molecules.^46–49^ At present, experimental insight is quite limited, in particular when it comes to the spatial range and frequency spectrum of these electric forces.

On a femtosecond time scale shorter than the breaking and reformation times of hydrogen bonds, the spectral diffusion of vibrational transition frequencies is a direct manifestation of fluctuating intermolecular electric forces.^40^ The time evolution of 2D infrared spectra has allowed the extraction of the FFCFs of water vibrations^50,51^ and—to lesser extent—of biomolecular vibrations^34,43,44^ which reflect the time scales and, thus, frequency spectrum of the fluctuating electric forces. In the bulk of a hydration shell at ambient temperature, the power spectrum of the intermolecular forces extends up to some 600 cm^−1^ or 18 THz.^52^ The spectrum is determined by the thermally excited intermolecular modes up to the L2 librations of (bulk) water.^52^ High frequency librations make a prominent contribution to the initial sub-100 fs decay of the bulk water FFCF.^50^ At the highly heterogeneous biomolecular surface, the motions of water molecules are slowed down compared to the bulk but—apart from practically immobilized waters placed in grooves or clefts—the extent of this slowing down is limited. Both 2D experiments and MD simulations^45^ give initial decay components of the FFCF on the order of hundreds of femtoseconds, corresponding to an average slowing down by less than a factor of 5. Such direct results—which do not depend upon MD—are inconsistent with the concept of an extremely slow hydration shell consisting of “biological water” which has been sometimes inferred from, e.g., time-resolved solvation studies.

Electric field amplitudes at biomolecular surfaces have been derived from MD simulations,^45,49^ static measurements of vibrational Stark shifts,^53,54^ and the analysis of 2D infrared spectra.^32^ In most cases, time-averaged values from several tens to 100 MV/cm have been found, with relative fluctuation amplitudes on the order of ±30%. Water molecules in the hydration shell represent the main sources of these fields, with some additional but limited contributions of ionic groups and/or counterions. The electric fields' spatial range is limited to a few water layers and is typically less than 10 Å. Two-dimensional infrared spectra of DNA at different hydration levels suggest that the electric field at the DNA surface originates mainly from the first 2 water layers while the contribution of counterions is minor,^32^ a picture that is supported by MD simulations for model systems.^49,55^

#### 2. Hydrogen-bond and reorientation dynamics

A group of slower processes within the hydration layer involve rotational and hydrogen bond dynamics of water molecules on a picosecond time scale. The reorientation and the associated breaking and reformation of hydrogen bonds generate picosecond structure fluctuations and have a direct impact on the residence times of water molecules in the first hydration layer.^56^ Due to the heterogeneous character of a hydrated biomolecular surface, one finds a broad distribution of rotational relaxation times, hydrogen bond lifetimes, and residence times;^21,56,57^ in the time-correlation function of the orientation of water molecules, the heterogeneity leads to a pronounced non-exponential decay kinetics.^21,56,57^ Hydrogen bond lifetimes cover a range from approximately 1 ps for water-water H-bonds in the bulk up to tens of picoseconds for waters under steric constraint and/or interacting with functional groups of the biomolecule.^21,56,57^ The molecular motions underlying such dynamics in a broad range of molecular geometries are well described by the fundamental jump model.^58^

**FIG. 5.**
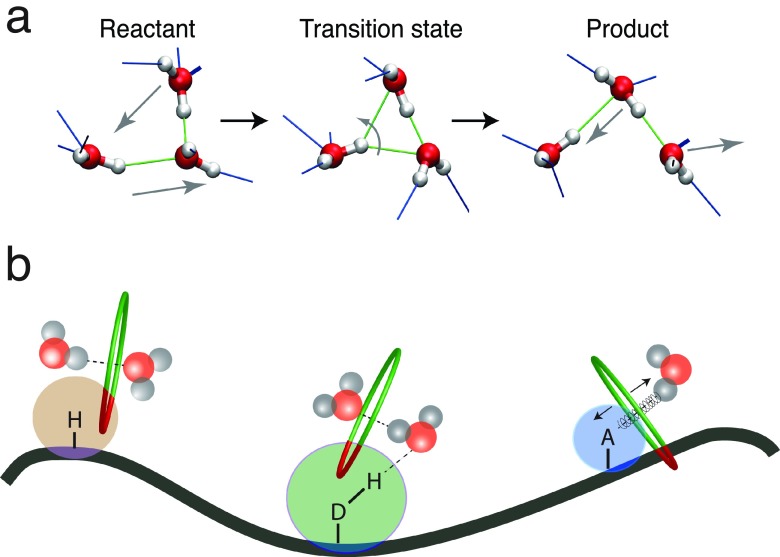
(a) Molecular jump mechanism for water reorientation. (b) Schematic figure with a protein interface and the three types of sites, respectively, hydrophobic, H-bond donor, and H-bond acceptor, together with a pictorial representation of the types of perturbation they induce on water dynamics (excluded volume and H-bond strength factors).

The jump mechanism involves sudden, large-amplitude jumps of a water molecule when a water hydroxyl (OH) group trades H-bond acceptors (Figure 5(a)).^58,59^ This concerted trading of H-bond acceptors can be regarded as a chemical reaction—complete with transition state—whose kinetics are determined by a free energy barrier due both to the approach of the new acceptor and to the elongation of the initial H-bond.^60,61^ The effect of a solute interface on the water H-bond dynamics can then be determined via the solute's impact on these two coordinates. The water jump rate constant depends on two key local solute features which reflect the solute's topographical and chemical aspects.^58^

The first feature is related to the solute's interface topography. It occurs for any type of solute and interface and is a consequence of the partial hindrance of the approach of a new water H-bond partner compared to the bulk situation. This produces a jump rate slowdown, quantified by the transition-state excluded volume factor *ρ_V_*, an activation-entropy effect.^62^ A water molecule next to a locally convex solute site experiences a slowdown of typically less than 2, while for a water molecule in a concave pocket this steric slowdown factor usually exceeds 2.^62^

The second feature is determined by the initial H-bond strength in the water's H-bond complex.^63^ Compared to the bulk situation, it accelerates (decelerates) the jump rate if the initial bond is weaker (stronger) than a water-water H-bond; this is quantified by the transition state H-bond strength factor $\rho_{\text{HB}}$. This effect can have both an enthalpic origin and an entropic origin when the initial H-bonded pair is held together by the shape of the local interface.

Each feature provides a quantitative, multiplicative factor affecting the jump rate.^58^ The reaction rate's overall perturbation factor relative to the bulk is then the product $\rho =\rho_{V}\rho_{\text{HB}}$. Figure 5(b) schematically summarizes the key effects expected for the three main types of sites found at a biomolecular interface: hydrophobic groups, H-bond donors, and H-bond acceptors. Hydrophobic groups only affect H-bond jump dynamics by hindering a new water H-bond acceptor's approach through an excluded volume effect. H-bond donors and acceptors act differently: the donors can form bonds of different strengths, but these bonds essentially act only on the water oxygen around which the angular jump occurs. The resulting torque's influence on the OH reorientation thus is negligible, and H-bond donors perturb water dynamics mainly via their excluded-volume effect. On the other hand, H-bond acceptor groups can impact water H-bond dynamics via both their excluded volume effect and the strength of the H-bond formed with water.

The jump model has been applied to biomolecules, including protein,^9,56,57^ DNA,^21^ and phospholipid bilayer^64^ hydration layers. Large angular jumps were shown to remain the main reorientation mechanism for water in the vicinity of biomolecules and the model-predicted water reorientation time distribution is in good agreement with that directly computed.^56^ For the very large fraction of water molecules within globular protein or DNA hydration shells that are moderately retarded with respect to the bulk (slowdown factor <2–3), the jump model suggests that this slowdown is an excluded-volume effect, due to the local topography of the biomolecule's interface which hinders the H-bond rearrangements.^9,21,56,57^ The slowdown factor scales inversely with the solvent-exposure of the site, and the width of the peak in the slowdown factor distribution arises from the corrugated biomolecular interface. The entropic nature of this steric slowdown is further supported by the weak temperature dependence of the average slowdown measured for the entire hydration shell for proteins by magnetic relaxation dispersion^65^ and reproduced in MD simulations.^9^ For DNA, the smaller peak at intermediate slowdown factor values (4 <ρ< 10) was shown to arise from water molecules next to the phosphate backbone.^9^ These sites are well solvent-exposed, so that steric effects are minimal; it is the strong H-bond with the phosphate sites that causes the observed slowdown.

The jump analysis indicates that an essential part of the effect induced by a biomolecule on its hydration shell arises from its local topography. However, a biomolecule is certainly not a rigid object and its conformation continually fluctuates, thereby inducing fluctuations of the local hydration dynamics. For example, in any one given site the water jump rate can change with the biomolecular conformation. Two limits can be considered here.^21^ In the first, water dynamics are much faster than the relevant dynamics of the biomolecule. Here, there is an inhomogeneous distribution of systems with different water dynamics. In the other, second extreme limit, the biomolecule samples its different local conformations before the hydration shell rearranges; here, the resulting water dynamics are equilibrated to and are averaged over these conformations, without displaying temporal heterogeneity. It was shown that the susceptibility of hydration dynamics vis-a-vis conformational fluctuations is enhanced in confined sites,^21^ e.g., in the DNA minor groove where a small widening of the groove can significantly accelerate the water dynamics. In these sites, the water and DNA groove dynamics occur on a similar time scale, thus showing that the biomolecule cannot be considered to be in either of the above limiting regions, i.e., neither static nor in equilibrium with the hydration layer dynamics. This is therefore in stark contrast with suggestions that the dynamics of biomolecules are slaved to their hydration shell.^66^

#### 3. Exchange of internal water molecules

We finally comment on the very much slower dynamics of water molecules inside a biomolecule, e.g., populating cavities in the protein interior. These internal water molecules are an integral part of the protein structure and can only exchange with the bulk with the aid of rare protein structural fluctuations on a time scale ranging from micro- to milliseconds. A recent effort combining NMR data and simulations has provided an improved understanding of the mechanism allowing the formation of transient water chains connecting these internal water molecules to the bulk.^67,68^

## III. CONCLUDING REMARKS

As recounted within, the evidence indicates that—although there is a large variety of biomolecular systems, each with its peculiar structures and properties—the water dynamics around these systems are governed by molecular mechanisms that are similar to those in bulk water, but are modified by the steric, electric, and other boundary conditions set by the biomolecule. In particular, the molecular mechanisms that explain the dynamics of water in the vicinity of biomolecules are in the main the same as those operative for water next to, e.g., polymers, ions, or small solutes. In this sense, biomolecular hydration water is not a distinct species and any label such as “biological water” is definitely inappropriate.

In our view, the main features of water structure and dynamics around biomolecules can be summarized as follows.

First, the biomolecular hydration shell should not be considered a homogeneous entity. The water molecules' arrangement and local interactions, most especially in the biomolecule's first layer, are strongly influenced by the heterogeneous geometric and chemical character of the biomolecular surface. As a result, water interactions and dynamics at protein, DNA, and other biomolecular interfaces are highly heterogeneous.

Second, the spatial range of the effect induced by the biomolecule on the surrounding water molecules is fairly short-ranged. The hydration shell assumes the structure and dynamics of bulk-like water within a few layers from the biomolecular interface, typically within fewer than five layers. Claims of a significant, much longer-ranged modification of the structure and dynamics of water around biomolecules lack theoretical and experimental evidence and are not supported by experiments mapping time-averaged or transient water structure and dynamics.

Biomolecular hydration shell dynamics covers a broad range of time scales. The fastest structural fluctuations in the first few water layers occur within a few hundred femtoseconds and are mainly related to water's librational degrees of freedom. The slower water reorientations typically take place on a timescale ranging from the ≃2 ps bulk value to several tens of picoseconds. This time scale arises primarily from hydrogen-bond network rearrangements via large-amplitude jumps executed by water molecules when they exchange hydrogen-bond partners. The slowing down of these water structural fluctuations is usually found to be moderate, i.e., on average less than a factor of five compared to bulk water; important exceptions with more marked (typically 10 to 50-fold^21^) slowdowns are for those special water molecules with marked spatial constraints, such as water located in the minor groove of DNA (which are strongly coupled to the DNA groove width dynamics^21^) and in clefts and pockets of protein surfaces. Returning to the typical case, this slowing down arises from the retardation induced by the biomolecular interface on the hydrogen-bond jumps, mostly by a steric factor due to the hindering by the interface of the approach of a new acceptor, and also by an enthalpic factor arising from the strength of the water-biomolecule hydrogen-bond to be broken. For example, the very slow dynamics measured in TDSS experiments is thus not due to intrinsically slow water molecules within the biomolecular hydration shell but rather originates from slow biomolecular motions,^69,70^ sometimes induced by the chromophore's presence,^71^ which can displace water molecules on a very slow time scale.

The electric fields at the interface between a biomolecule and its water shell can reach values up to 100 MV/cm. The water molecules in the first two hydration layers are a major source of such fields, whose range is limited to a sub-10 Å length scale. Amplitude fluctuations of these local electric fields of up to several tens of MV/cm can be produced by the water shell's (and the biomolecule's) structural fluctuations and cover a very broad frequency spectrum up to terahertz frequencies.

The pioneering TDSS experiments by Ahmed Zewail and his co-workers have been instrumental for developing new experimental and theoretical concepts for gaining insight into biomolecular hydration dynamics at the short time scale of molecular motions. Future experimental progress requires the application of probes combining spatial selectivity with femto- to picosecond time resolution, in order to map the spatio-temporal dynamics of hydrated biomolecules, preferably in a non-invasive way. Of particular relevance in this connection are structure-sensitive methods such as time-resolved x-ray diffraction, scattering, and absorption, complemented by multidimensional optical spectroscopy. In the areas of the theory and simulation, the effect of a biomolecule on the structure and dynamics of individual water molecules appears to be now reasonably well understood. Some pressing challenges here will be to extend (or replace) these models in order to describe any important collective dynamics that may exist and, most importantly, to describe more physiologically relevant situations. For example, in the crowded cellular environment, water molecules are simultaneously affected by several biomolecules and ions. New developments will be required to understand the interplay between these different non-additive effects contributing to experimental dynamical effects.^25^
